# Long-read sequencing reveals increased isoform diversity in key transcription factor effectors of intercellular signalling at the invertebrate-vertebrate transition

**DOI:** 10.1186/s12915-026-02522-w

**Published:** 2026-01-24

**Authors:** Nuria P. Torres-Aguila, Marika Salonna, Sebastian M. Shimeld, Stefan Hoppler, David E. K. Ferrier

**Affiliations:** 1 The Scottish Oceans Institute, School of Biology, University of St Andrews, St Andrews, Fife KY16 8LB UK; 2 Current address: Departament de Genètica, Microbiologia I Estadística, Facultat de Biologia, Institut de Recerca de La Biodiversitat (IRBio), Universitat de Barcelona, Barcelona, 08028 Spain; 3 Institute of Medical Sciences, Foresterhill Health Campus, University of Aberdeen, Aberdeen, AB25 2ZD UK; 4 Current address: School of Molecular Biosciences, College of Medical, Veterinary & Life Sciences, University of Glasgow, Glasgow, G12 8QQ UK; 5 The Department of Biology, University of Oxford, Life and Mind Building, South Parks Road, Oxford, OX1 3EL UK

**Keywords:** TCF, SMADs, GLIs, *Ciona*, Lamprey, *Lampetra planeri*, *Xenopus tropicalis*, Splicing

## Abstract

**Background:**

Several intercellular signalling pathways (including wingless (Wnt), hedgehog (Hh), and bone morphogenetic protein (BMP)) are used repeatedly in animals throughout development and evolution and are also frequent targets for disease-associated disruptions. We have previously shown that the major transcriptional effectors of β-catenin-dependent Wnt signalling, the TCF/LEF proteins, in contrast to other pathway components, have a higher gene number and isoform diversity in vertebrates versus invertebrates, but this increased diversity has only been poorly quantified. Considering that isoform diversity correlates with organism complexity, any increase in major signalling effectors is likely to have made a significant contribution to vertebrate evolution.

**Results:**

Using de novo long-read transcriptomes, we compared isoform number per gene for the chordates *Ciona intestinalis*, *Lampetra planeri* and *Xenopus tropicalis*, thus encompassing the invertebrate sister group to vertebrates, as well as a cyclostome and a gnathostome vertebrate. We find a significant increase in the number of transcript isoforms per gene expressed during embryo development and organogenesis at the invertebrate-to-vertebrate transition, specifically for the main transcription factor effectors of the Wnt/β-catenin, Hh and BMP pathways, i.e. TCF/LEF, GLI and SMAD.

**Conclusions:**

Our results implicate an increase in isoform diversity of the transcription factors of major intercellular signalling pathways as having a disproportionate role in the evolutionary origin and diversification of vertebrates.

**Supplementary Information:**

The online version contains supplementary material available at 10.1186/s12915-026-02522-w.

## Background

The driving forces for the evolution of organism complexity has been a topic of discussion for decades [1–4]. Despite genome duplications being renowned for the creation of new paralogous gene copies and their subsequent evolution via processes like sub- and neofunctionalisation [5], and specialisation [6], the G-value paradox showed that the number of genes in a genome do not necessarily correlate with organism complexity [2]. One of the proposed alternatives to solve this paradox is the expansion of the organism proteome through alternative splicing, correlating with phenotypic novelty [3]. Previous studies found a strong correlation between number of cell types (as a proxy for organism complexity) and alternative splicing [4, 7], providing evidence of the importance of isoform diversity for organism evolution. However, whether particular types of genes contribute disproportionately to this phenomenon has not been assessed.

Wnt signalling is a cell-to-cell signalling mechanism highly conserved in the animal kingdom and required during development and regeneration [8]. The best described Wnt pathway is the canonical Wnt (cWnt) pathway, also known as the Wnt/β-catenin pathway, which involves the nuclear translocation of β-catenin, triggered by extracellular Wnt ligand-receptor interactions. Nuclear β-catenin functions as a co-regulator for activation of Wnt-target genes, usually via binding the T-cell factor/lymphoid enhancer factor (TCF/LEF) proteins. The cWnt pathway has a variety of roles in animal homeostasis and development, including involvement in development of the anterior–posterior and dorsal–ventral axes [8, 9]. It is also associated with many human diseases such as cancers [10], diabetes [11] and mental disorders [12]. Comparably widespread functions in development, homeostasis and disease are also seen in other major signalling systems such as the hedgehog (Hh) and bone morphogenetic protein (BMP) pathways, whose main transcription factors are the Glioma-Associated Oncogene (GLI) proteins and the small/Mothers Against DPP Homolog (SMAD) proteins, respectively [13–15].

Genome comparisons between vertebrates and invertebrates reveal a remarkable conservation of the cWnt pathway with relatively little expansion of most of its components [16]. Nonetheless, vertebrate TCF/LEF transcription factors, the main transcription factor of the cWnt pathway, show a much greater diversity [17–20]. Multiple copies of *TCF/LEF* genes have been retained from genome duplications in vertebrates, which typically possess four TCF/LEF family genes with multiple isoforms, while invertebrates typically have one *TCF* gene with a single isoform [20, 21]. A similar gene expansion might have occurred for SMAD and GLI families of transcription factors mediating BMP/TGFβ and Hh signalling, respectively.

Given these general observations, we aimed to assess transcript isoform diversity of developmentally expressed genes across components of these signalling pathways (cWnt–TCF/LEF, BMP–SMAD, Hh–GLI) and compare them to other categories of genes. We hypothesised that such major developmental control genes may have been a particular target for the evolutionary diversification that occurred with the origin of the vertebrates. We selected three species representing key lineages of the Olfactores chordates; the invertebrate urochordate *Ciona intestinalis*, the cyclostome (jawless vertebrate) *Lampetra planeri*, and a gnathostome (jawed vertebrate) *Xenopus tropicalis*, to analyse in an unbiased way the number of genes and transcripts expressed during embryogenesis and assess if TCF/LEF, SMAD and GLI genes are distinctive in their transcript isoform diversity. Our use of de novo long-read sequencing data was focused specifically on selected developmental stages of the three chosen species to help overcome the difficulty in accurately and reliably determining splice isoforms from short-read sequencing data. Also, our approach allows us to determine which genes and isoforms are specifically deployed during development to improve the comparability of our data between species.

## Results

### Transcriptome analysis

**Fig. 1:**
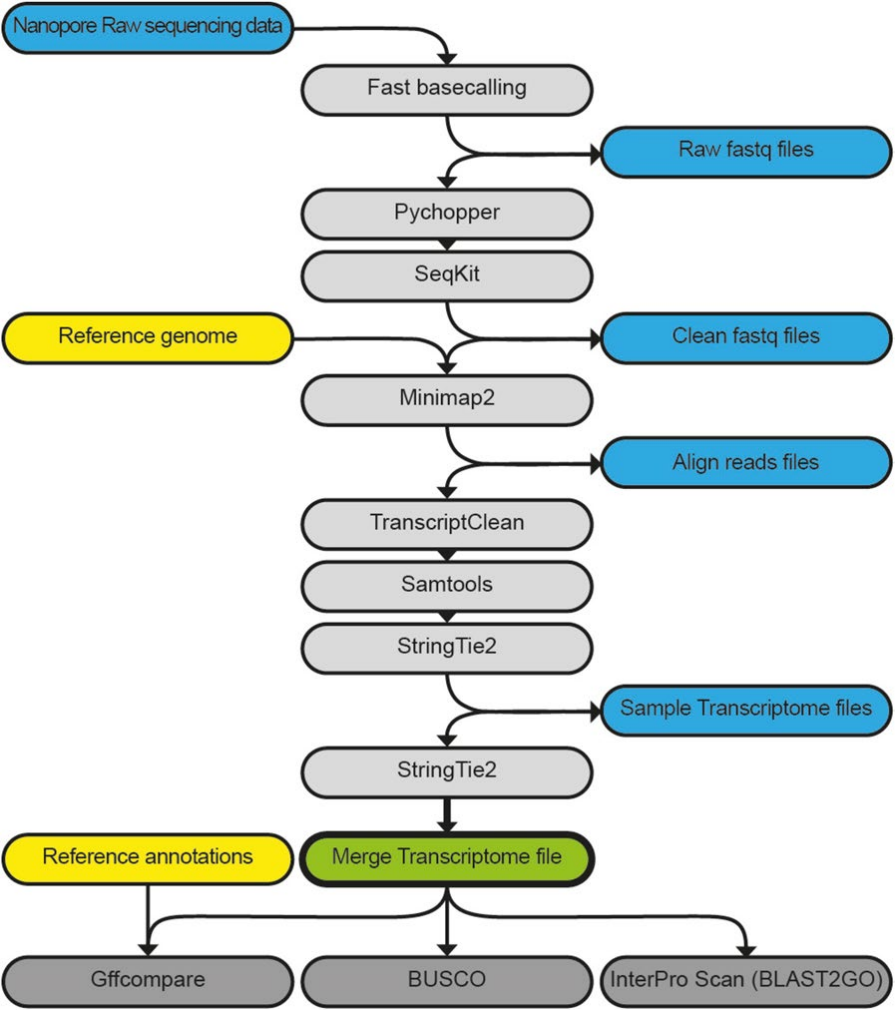
Pipeline for long-read data processing. Round boxes: files; square boxes: software programmes. Blue boxes: obtained files; yellow boxes: reference files; green box: final transcriptome file; light-grey boxes: software for processing data; dark-grey boxes: software for evaluating quality of obtained transcriptome file

**Fig. 2:**
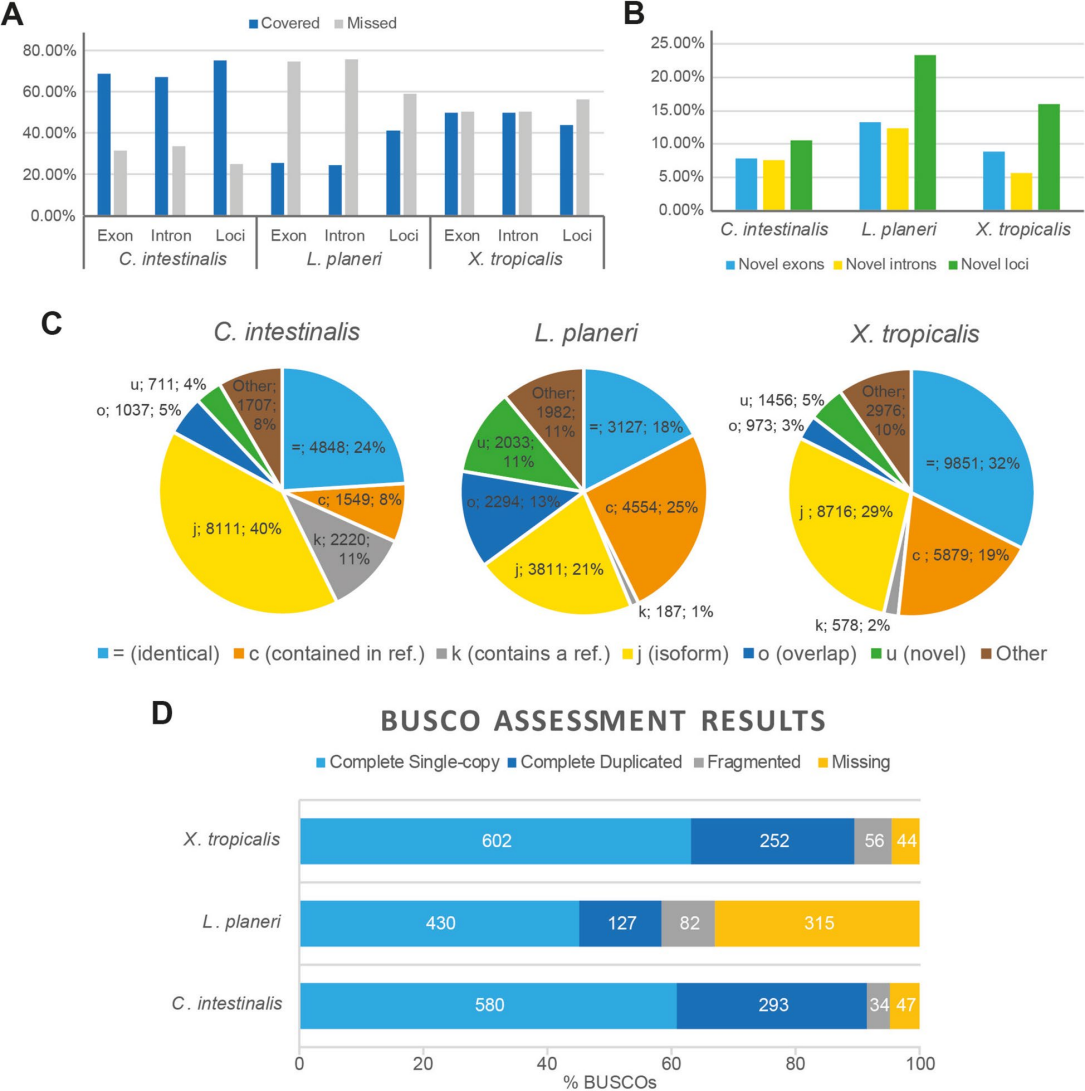
Transcriptome quality assessments. **A** Covered (blue) and missed (grey) exons/intron/loci for each species data set. **B** Novel exon (blue), intron (yellow), and loci (green) for each species data set. **C** Pie plots showing the distribution of transcript-matching types according to gffcompare categories: = (light-blue), identical reference-query match; c (orange), complete query match within reference; k (grey), complete reference match within query; j (yellow), some splice site mismatch (potential new isoform); o (dark-blue), partial overlapping match; u (green), no match of query within reference (novel); Other (brown), other types of query-reference matches including m (all introns retained), n (some introns retained), e (single exon match), s (intron match on opposite strand), x (exon match on opposite strand), i (contained within reference intron), y (reference contained within intron), p (possible polymerase run-on), and r (repeat). **D** Histogram showing the amount of evolutionary conserved orthologues (BUSCOs) found, as single-copy (light blue), duplicated (dark blue) or fragmented (grey), and missing (yellow)

To analyse the diversity of isoforms of developmentally transcribed genes within chordates, we performed cDNA long-read sequencing of developmental stages of *C. intestinalis*, *L. planeri* and *X. tropicalis* (see Table 1) and processed the data following the pipeline shown in Fig. 1. All selected stages performed similarly in the sequencing protocol (Table 1) producing de novo transcriptomes with loci coverage over 40% (Fig. 2A) and capturing over 60% of metazoan-conserved orthologues (BUSCOs) (Fig. 2D). Notably, the transcriptomes included over 10% of novel loci, with gene models not currently annotated in the respective reference genomes (Fig. 2B), although the highest proportion of transcripts were ones that fully matched reference models (categories ‘ = ’, ‘c’ and ‘k’ of Fig. 2C). Regarding the novel loci, the majority of the identified genes had no GO term associated with them (1110 out of 1359 for *C. intestinalis*, 3184 out of 3337 for *L. planeri*, and 2898 out of 3316 for *X. tropicalis*) (see the ‘Methods’ for GO analysis details). This suggests these novel loci that lack associated GO terms may be taxon-specific or rapidly evolving genes. After performing a GO enrichment analysis on the loci that did have associated GO terms, no GO terms were enriched for *C. intestinalis* or *L. planeri*. However, for *X. tropicalis* we found 209 GO terms significantly enriched (Additional File 1: Table S1), most of them linked to muscle- and heart-related functions, including muscle contraction, structural assembly, and development.

**Table 1:** Long-read sequencing reads. Reads obtained after long-read sequencing and after data processing for each sample used. Ci: *Ciona intestinalis*; Lp: *Lampetra planeri*; Xt: *Xenopus tropicalis*; St: developmental stage

Stage	Dev process	Input reads	Clean reads	Min. length	Avg. length	Max. length	Sum. length
Ci_St04	Cleavage	1,466,765	574,175	200	512	4385	293,959,329
Xt_St06	Cleavage	1,615,386	1,374,219	200	897.8	5474	1,233,804,871
Ci_St12	Gastrulation	2,807,191	2,156,039	200	891.2	7527	1,921,535,475
Xt_St10	Gastrulation	2,876,070	2,277,830	200	626.7	3851	1,427,433,920
Ci_St15	Neurulation	860,144	610,643	200	515.4	3441	314,711,689
Ci_St16	Neurulation	1,378,599	839,501	200	668.8	3560	561,488,108
Xt_St16	Neurulation	1,436,005	1,258,985	200	803.8	4049	1,011,971,517
Xt_St20	Neurulation	2,435,888	2,048,749	200	704.8	4483	1,443,978,930
Lp_St22	Post-neurulation	985,970	730,948	200	680.3	5565	497,288,356
Ci_St21	Cell Diff	3,526,232	2,724,082	200	647.3	3230	1,763,334,732
Ci_St26	Cell Diff	2,601,133	1,913,539	200	566.5	4336	1,083,927,850
Lp_St25	Organogenesis	7,505,549	4,919,531	200	536.8	4812	2,640,918,572
Lp_St28	Organogenesis	5,515,552	3,719,539	200	534.1	5,287	1,986,704,869
Xt_St28	Organogenesis	1,871,477	1,424,131	200	555.6	4628	791,301,969
Xt_St35	Organogenesis	2,506,733	1,541,432	200	440.9	2996	679,623,175

**Fig. 3:**
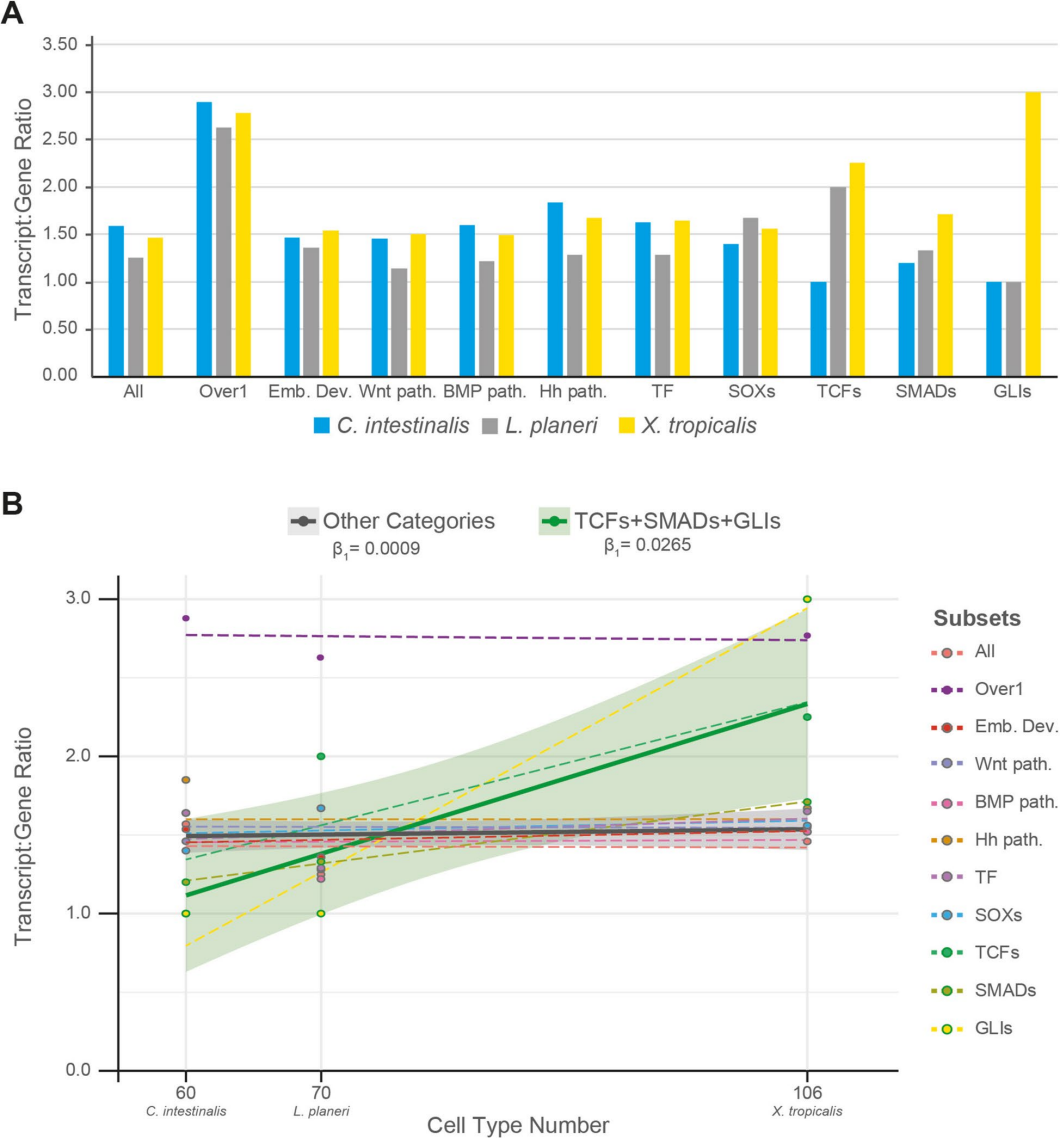
Transcript:gene ratio. **A** Histogram of the transcript:gene ratios (t/g ratio) for each species (blue: *Ciona intestinalis*; grey: *Lampetra planeri*; yellow: *Xenopus tropicalis*) and category (All: whole transcriptome; Over 1: genes with more than one transcript; Emb. Dev: genes with the GO term ‘Embryo Development’ (GO:0009790); Wnt path.: genes with the GO term ‘Wnt signalling pathway’ (GO:0016055); BMP path.: genes with the GO term ‘BMP signalling pathway’ (GO:0008101 or GO:0030509); Hh path.: genes with the GO term ‘smoothened signalling pathway’ (GO:0007224); SOXs: *SOX* genes; TF: genes with the GO term ‘DNA-binding transcription factor activity’ (GO:0003700); TCFs: *TCF* and *TCF/LEF* genes; SMADs: *SMAD* genes; GLIs: *GLI* genes). **B** Linear regressions of each subset studied (dotted lines) and for the two groups used for the Mann–Whitney *U* test (solid lines): ‘Other categories’, encompassing All, Emb. Dev., Wnt path., BMP path., Hh path., TF and SOXs (grey line), and ‘TCFs + SMADs + GLIs’, encompassing those three gene families (green line). The t/g ratio is plotted against the cell type number in development for each species, according to Cao et al*.*^46^ for *Ciona*, Pang et al*.*^47^ for lamprey, and Liao et al.^48^ for *Xenopus*. Dots: Transcript:Gene ratio values. Dot outline: belonging to group, ‘TCFs + SMADs + GLIs’ (green) or ‘Other categories’ (grey). β_1_: regression coefficient

The transcript:gene ratio (t/g ratio) was calculated for the total number of expressed genes and transcripts obtained for each transcriptome, as well as for subsets of particular gene categories (Table 2). We observed a higher number of different transcripts per expressed gene in the vertebrates relative to the invertebrate *Ciona* only for TCF/LEF genes (TCFs), SMADs and GLIs (Fig. 3A). Comparisons of the variance of t/g ratio observed on each subset showed that TCFs had a greater variance than most of the other subsets studied (*p*-value < 0.1, Additional File 1: Tables S2–5), including the subset ‘Wnt signalling pathway’ (GO:0016055) and the SOX genes, which belong to the same HMG-box superfamily as the TCF/LEFs. Moreover, a similar pattern was found for SMAD and GLI genes. However, after multiple-test correction, none of these comparisons remained significant (Additional File 1: Table S2). To further assess whether the increase in transcripts observed for these gene families was different from that observed for the other categories, we performed linear regressions for each subset and calculated the regression coefficient (β1 or slope, Fig. 3B, Additional File 1: Tables S6–8), excluding the t/g ratio of the target gene families from the t/g ratio calculations on the other categories (i.e. All, Over1, Emb. Dev., Wnt path., BMP path., Hh path., and TF, Additional File 1: Table S2). A linear regression model including all individual subsets revealed that the interaction term of GLIs (cell_type_num:SubsetGLIs) and TCFs (cell_type_num:SubsetTCFs) showed significance (*p*-value < 0.01 and *p*-value < 0.1, respectively, Additional File 1: Table S6), indicating that the regression coefficient observed for those subsets differed from the reference subset (All).

**Table 2:** Total number of genes and transcripts for each transcriptome and subsets. All: the whole transcriptome; Over 1: only genes with more than one transcript; Emb. Dev.: genes with the GO term ‘Embryo Development’ (GO:0009790); Wnt Path.: genes with the GO term ‘Wnt Pathway’ (GO:0016055); BMP Path.: genes with the GO term ‘BMP signalling pathway’ (GO:0008101 or GO:0030509); Hh Path.: genes with the GO term ‘smoothened signalling pathway’ (GO:0007224); TF: genes with the GO term ‘DNA-binding transcription factor activity’ (GO:0003700); SOXs: *SOX* genes; TCFs: *TCF* and *TCF/LEF* genes; SMADs: *SMAD* genes; GLIs: *GLI* genes

	***Ciona intestinalis***			***Lampetra planeri***			***Xenopus tropicalis***		
	**Genes**	**Transcripts**	**Ratio t/g**	**Genes**	**Transcripts**	**Ratio t/g**	**Genes**	**Transcripts**	**Ratio t/g**
**All**	12,717	20,183	1.59	14,311	17,988	1.26	20,775	30,429	1.46
**Over 1**	3868	11,171	2.89	2235	5860	2.62	5431	15,105	2.78
**Emb. Dev**	15	22	1.47	14	19	1.36	69	106	1.54
**Wnt Path**	40	58	1.45	29	33	1.14	89	134	1.51
**BMP Path**	54	86	1.59	37	45	1.22	63	94	1.49
**Hh Path**	49	90	1.84	43	55	1.28	49	82	1.67
**TF**	296	481	1.63	130	167	1.28	750	1235	1.65
**SOXs**	5	7	1.40	3	5	1.67	9	14	1.56
**TCFs**	1	1	1.00	1	2	2.00	4	9	2.25
**SMADs**	5	6	1.20	3	4	1.33	7	12	1.71
**GLIs**	1	1	1.00	1	1	1.00	1	3	3.00

Next, we performed separate linear regression models for each gene family (TCFs, SMADs, GLIs and SOXs), grouping the remaining subset to increase statistical power. We excluded the Over1 subset, as its high mean increased the regression error, and the pathways subsets not related with the specific gene family, except for SOXs (i.e. TCFs groups: TCFs and All + Emb.Dev. + Wnt path. + TF + SOXs; SMADs groups: SMADs and All + Emb.Dev. + BMP path. + TF + SOXs; GLIs groups: GLIs and All + Emb.Dev. + Hh path. + TF + SOXs; SOXs groups: SOXs and All + Emb.Dev. + Wnt path. + BMP path. + Hh path. + TF). The interaction terms showed that TCFs, SMADs, and GLIs had a significantly different regression coefficient compared to the grouped subsets (TCFs *p* < 0.01; SMADs *p* < 0.1; GLIs *p* < 0.001, see Additional File 1: Table S7) whereas SOXs showed no significance (*p*-value > 0.1; Additional File 1: Table S7). This reaffirmed the previously observed pattern for GLIs and TCFs and extended it to SMADs.

Finally, we performed a linear regression model grouping TCFs, SMADs, and GLIs together as target group, and the remaining subsets (i.e. All, Emb. Dev., Wnt path., BMP path., Hh path., TF and SOXs) as comparison group. The regression coefficient observed for the target group was significantly higher than the one observed for the comparison group (Fig. 3B, Shapiro–Wilk test *p*-values > 0.5; Bartlett test *p*-value < 0.001; Mann–Whitney *U* test: *p*-value < 0.05, Additional File 1: Table S8).

All together, these results indicate that these key developmental transcription factors (TCF/LEFs, SMADs, and GLIs) have a distinctive pattern of a higher number of different transcripts per expressed gene in the vertebrates relative to the invertebrate *Ciona*: in our analysis (summarised in Table 2), TCF/LEFs have 1 gene and 1 transcript in *C. intestinalis* compared to 1 gene and 2 transcripts in *L. planeri* and 4 genes and 9 transcripts in *X. tropicalis*; SMADs have 5 genes and 6 transcripts in *C. intestinalis* compared to 3 genes and 4 transcripts in *L. planeri* and 7 genes and 12 transcripts in *X. tropicalis*; and GLIs have 1 gene and 1 transcript in *C. intestinalis* compared to 1 gene and 1 transcript in *L. planeri* and 1 gene and 3 transcripts in *X. tropicalis* (DNA and Protein sequences available in Additional Data 1–6). Interestingly, the variance observed in the t/g ratio for these three gene families (TCF/LEFs, SMADs, and GLIs) was not significantly different between each of them (padj = 1, Additional File 1: Table S2), indicating that they show similar distributions of transcript isoforms per expressed gene within the three chordate groups. Alongside the linear regression modelling that demonstrates a significant increase in t/g ratios of the developmentally expressed genes in the TCF/LEF, GLI and SMAD families in the two vertebrates relative to the invertebrate *Ciona*, this demonstrates a distinct characteristic of these families relative to other genes found in our transcriptome data.

### A new splice isoform in *Ciona intestinalis*

To confirm the transcript sequence and structure of the *C. intestinalis* TCF/LEF (in what was formerly known as *Ciona intestinalis* Type B) relative to the commonly studied sister species *Ciona robusta* (formerly *Ciona intestinalis* type A), we performed RACE-PCR in different stages of development, selected according to the previously described expression of *C. intestinalis TCF* (*CiTCF*) [22]. 5′RACE-PCR was performed in St04 (8-cell; maternal mRNA) and St12 (mid-Gastrula; zygotic mRNA), and 3′RACE-PCR was performed for St04, St12, St16 (late Neurula), and St21 (mid-Tailbud I).

**Fig. 4:**
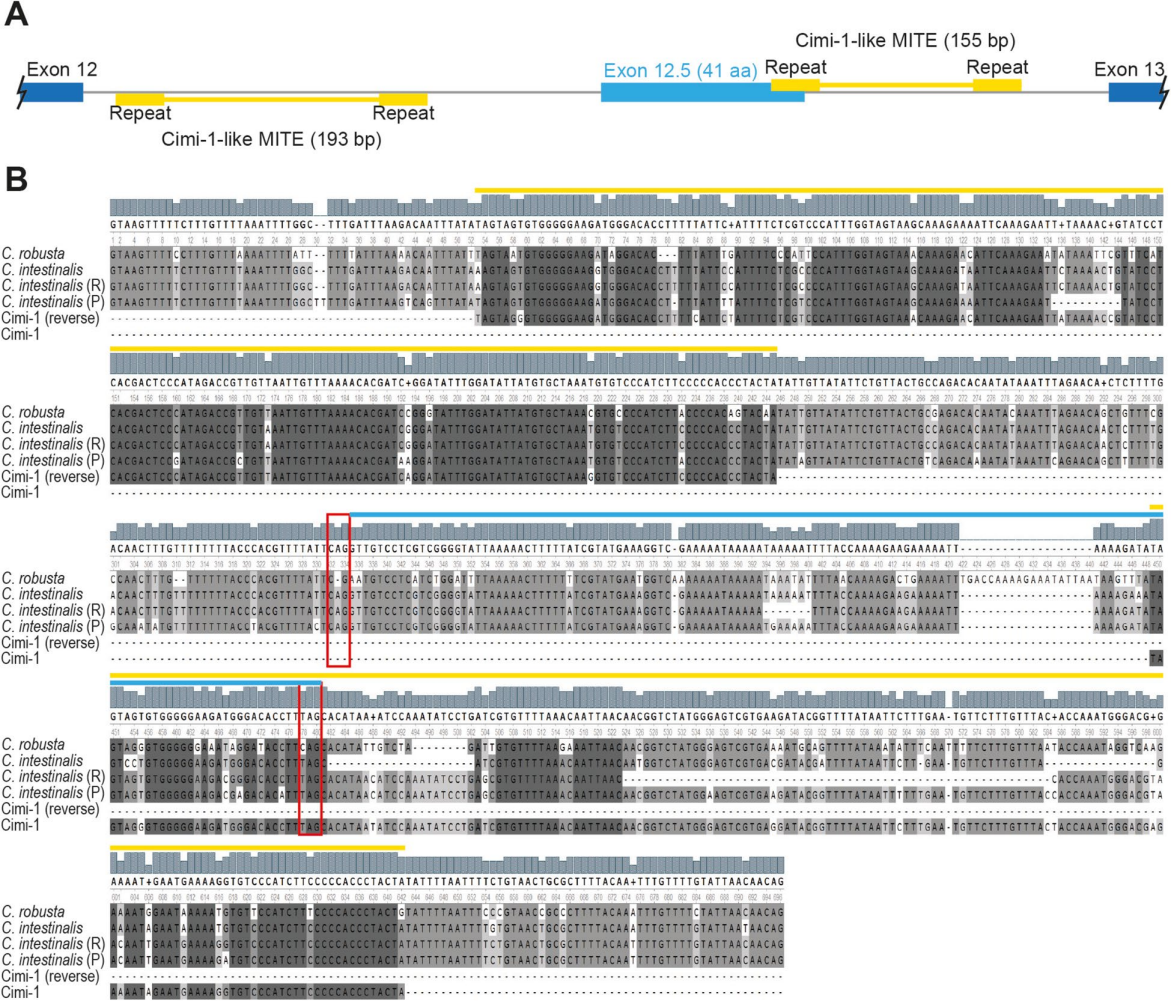
Schematic of *CiTCF* intron 12. **A** Schematic of the *C. intestinalis* genomic region where exon 12.5 is found. **B** DNA alignment of *TCF* intron 12 of *C. intestinalis* and *C. robusta*. *C. intestinalis* (R): sequence from Roscoff reference genome (GCA_018327825.1); *C. intestinalis* (P): sequence from Plymouth reference genome (GCA_018327805.1); dark blue: annotated exons; light blue: new exon (exon 12.5); yellow: Cimi-1-like sequences; red boxes: acceptor site and stop codon of exon 12.5

For 5′RACE-PCR, only one fragment was amplified, matching the described gene model. For 3′RACE-PCR two different 3′ ends were found. The first was found in all the assessed stages and matched the previously described gene model. The second, smaller in size, was found in St16 and matched the described gene model but with a different final exon. Further analysis of the genomic region between exon 12 and exon 13 in *C. intestinalis* (intron 12; Fig. 4A) showed the presence of this new exon flanked by two transposable elements, partially overlapping one at the 3′ end. These transposable elements matched in sequence the previously described miniature inverted-repeat transposable elements (MITE) Cimi-1 [23]. Comparison of intron 12 between *C. robusta* and *C. intestinalis* revealed that despite the Cimi-1 insertions being conserved, this was not the case for the splice acceptor site nor the stop codon of exon 12.5 (Fig. 4B), indicating that this newly discovered exon may be specific to *C. intestinalis*. To confirm this alternative C-terminus, we designed a specific Reverse primer for exon 12.5 and performed RT-PCRs on St12, St15 (mid-Neurula) and St21. This isoform was found only in post-gastrulation stages. *C. intestinalis* thus produces two isoforms of TCF/LEF, in contrast to the single isoform produced by this gene in *C. robusta*.

## Discussion

Our transcriptome analyses focused on gene expression during embryo development and organogenesis revealed that *TCF/LEF*, *SMAD* and *GLI* genes exhibit a distinctive pattern of higher numbers of splice isoforms per developmentally expressed gene in vertebrates than the representative from the closest invertebrate sister group, represented here by the urochordate *C. intestinalis*. This larger number of splice isoforms occurs in addition to the increase in paralogue numbers in these transcription factors at the origin of vertebrates.

Our previous in silico analyses identified an increase in TCF/LEF gene number encoded in the genome at the invertebrate-to-vertebrate transition, likely via the whole genome duplications that occurred early in vertebrate evolution, and in addition suggested there was an increase in isoform diversity in this gene family [21]. However, this diversity remained to be quantified. It also remained to be determined as to when these isoforms are expressed during development and hence are presumably functional. This analysis is provided here. Another important question was whether the evolutionary patterns seen in this major transcriptional effector of the cWnt signalling pathway were unique, or also occurred elsewhere, especially in other major developmental signalling pathways like Hh and BMP/TGFβ.

Invertebrates typically have four SMADs, corresponding to a single-copy of each SMAD subgrouping (common SMAD (Co-SMAD), inhibitory SMAD (I-SMAD), BMP-regulated receptor SMAD (R-SMAD) and TGFβ-regulated R-SMAD). However, *Ciona* has independently duplicated the TGFβ-regulated R-SMAD [24], giving a total of five SMADs in this tunicate. Meanwhile vertebrates usually possess eight different SMADs (one Co-SMAD, two I-SMADs, three BMP-regulated R-SMADs, and 2 TGFβ-regulated R-SMADs) [25, 26]. In the GLI family, invertebrates have a single *GLI* gene while vertebrates typically have three paralogues [27]. Thus, the SMAD and GLI families show a similar pattern of increased paralogue numbers encoded by the genome to that seen in the TCF/LEF family. However, the amount of developmentally expressed splice isoform diversity remained to be analysed for both SMAD and GLI families. Our data demonstrate that these families, when analysed by linear regression modelling against numbers of cell types in these chordates, show a similar pattern to the TCF/LEF family, with a significantly higher number of isoforms per developmentally expressed gene in the vertebrates we studied relative to the invertebrate sister group.

It is striking that this invertebrate-to-vertebrate pattern for the TCF/LEF, SMAD and GLI genes is significantly different from other developmentally expressed genes and their transcripts found in our new transcriptomes (‘All’ category in Fig. 3). This is also the case when we focus on genes that demonstrably exhibit alternative splicing (that is, have more than one transcript per gene: the ‘Over 1’ category in Fig. 3), in which the *C. intestinalis* ratio is indistinguishable from those of the two vertebrates. Thus, there is not simply a general increase in isoform diversity in developmentally expressed genes at the invertebrate-to-vertebrate transition. Since these first two categories encompass genes that span a variety of biological functions, we also focused on genes thought to be more specifically involved in embryo development, in case there is a general increase in complexity of developmental control genes associated with the invertebrate-to-vertebrate transition and the evolution of vertebrate complexity. No distinct pattern was observed between the invertebrate *C. intestinalis* and the vertebrates. This lack of invertebrate to vertebrate distinction was also observed when the focus was even more specific, onto Wnt pathways as a whole (Fig. 3). Another alternative possibility was explored by comparison to Transcription Factors in general (‘TF’ in Fig. 3) in case the mediators of transcriptional control are the focus of change between invertebrates and vertebrates. No significant distinction was found. As a final test of how distinct the pattern found for the TCF/LEF family was, we analysed the SOX genes, since these are in the same superfamily as the TCF/LEF genes and hence act as the closest comparison possible. The invertebrate-to-vertebrate pattern for the TCF/LEF genes is significantly different to that of the SOX genes. Thus, the TCF/LEF genes stand-out from all of these different categories of genes, implying a specific expansion in the splice isoform diversity focused on these transcription factor mediators of the cWnt pathway. Notably, the only categories of genes that we found with comparable invertebrate-to-vertebrate patterns were those of the transcription factor mediators of other major intercellular signalling pathways (the SMAD and GLI genes).

Amongst all of this vertebrate genetic diversity, it has been shown that vertebrate TCF/LEF paralogues and GLI paralogues have some degree of redundancy at the functional level [17, 28]. Nevertheless, there is also evidence that different TCF/LEF isoforms can target different genes [29], showing both sub- and neo-functionalisation. Similarly, GLI isoforms have been shown to have opposing roles activating or repressing the gene expression of their specific target genes [28]. Therefore, the diversity of vertebrate genes in TCF/LEF, SMAD and GLI families, and the isoforms produced from them, presumably reflects a wide array of functional capabilities downstream of important developmental signalling pathways in vertebrates.

These three gene families are the main transcription factor effectors of major intercellular signalling pathways (cWnt, BMP/TGFβ and Hh, respectively), which are integral to embryo development, organogenesis and homeostasis. Their major roles in development in conjunction with the correlation of isoform diversity with organism complexity [1–4] is consistent with the hypothesis that increased diversity of these transcription factors may be making a disproportionate contribution to the evolution of vertebrate complexity relative to invertebrates. Interestingly, previous studies had observed genes resulting from duplication usually retain lower numbers of isoforms, with duplication and isoform diversity being inversely correlated evolutionary patterns [30, 31]. This makes the pattern observed here in these developmental signalling transcription factor effectors even more striking, as our data shows that *TCF/LEF*, *SMAD* and *GLI* genes are exceptions to this inverse correlation. This could be an indicator of a significant role for isoform diversity of these key transcription factors in the evolutionary origins and diversification of vertebrate complexity.

One caveat to this hypothesis is whether the species selected here are good representatives of the invertebrate-to-vertebrate transition. *C. intestinalis*, for example, was selected because it is a urochordate and as such is a member of the closest invertebrate clade to the vertebrates, and is also accessible and amenable to gene expression and developmental experimentation. There are, however, aspects of its genome organisation and content that are relatively derived within the chordates [32]. Also, it is known that amphioxus exhibits alternative splicing from its *Gli* gene, producing two distinct isoforms [33], rather than the single isoform of *Ciona GLI* that was found here. It is also notable that our analyses are focused on the transcripts found within our new transcriptome data. While this enhances comparability between the different species, we are not necessarily capturing all isoforms produced by each of the three species selected. Rare transcripts expressed at very low levels in embryogenesis, or transcripts expressed only in adult stages, will not be present in our data. These are areas for future further work, to quantify the patterns described here with even greater precision. Nevertheless, there is no indication that the three transcription factor families focused on here are unusual in *Ciona* relative to invertebrates in general in any major way, but this is also an area for further scrutiny in the future, particularly as additional high-quality genome assemblies and more long-read transcriptome data becomes available.

In addition to these ‘signalling transcription factor’ findings, the long-read transcriptomes provided in this work are also a valuable resource for deeper understanding of gene expression during embryo development of different chordates, including species not previously assessed with long-read transcriptome sequencing, such as Cyclostomata and Urochordata. However, despite all the samples being processed in the same way and our obtaining similar quality values within urochordate and gnathostome data sets, the quality of the cyclostome data set was not as good (Fig. 2). This issue could be due to the GC-richness of cyclostome genomes [34] and/or the fact that *L. planeri* (the species sampled) and *P. marinus* (the species used as the lamprey reference genome) are more evolutionarily distant and distinct than the species used for urochordates (samples of *C. intestinalis* and reference genome of *C. robusta*) and gnathostomes (samples and reference genome of *X. tropicalis*).

It is also notable that for *C. intestinalis*, the transcript type category that had the highest number of transcripts was ‘j’ (40%, Fig. 2C), showing that most of the transcripts had mismatched splicing sites (junctions) against reference annotations. This could be an indicator of potential novel isoforms found in this new *C. intestinalis* transcriptome data for previously annotated genes in *C. robusta*. In fact, our finding of an alternative transcript of *CiTCF* is the first evidence that *C. intestinalis* has more than one TCF isoform. However, this second transcript of *CiTCF* was only found by RACE-PCR rather than being in the transcriptome data, which may reflect the RACE-PCR having higher sensitivity than cDNA long-molecule sequencing, since the PCR is gene-specific. In addition, the partial overlap of exon 12.5 with the Cimi-1 MITE sequence provides an example of how transposable elements can alter intronic sequences that then provide material for the evolutionary origin of novel exons, in this case in concert with point mutations that generated a new splice acceptor site as well as a new stop codon.

## Conclusions

We have created de novo transcriptomes of embryo development for three different chordates: *C. intestinalis* (Urochordata), *L. planeri* (Cyclostomata) and *X. tropicalis* (Gnathostomata). Our analyses demonstrate distinctive increases in isoform diversity at the invertebrate-to-vertebrate transition specifically among transcription factor effectors of key intercellular signalling pathways that drive cell type diversity. This distinctive change focused on these specific gene families (TCF/LEF, SMAD and GLI) goes beyond the previous observations of a general correlation between increased isoform diversity and evolution of animal complexity. This demonstrates likely disproportionate roles for these specific transcription factor families in the evolution of vertebrate complexity, which needs to be explored with future functional assays of these various isoforms.

## Methods

### Material fixation and RNA extraction

After in vitro fertilisation, selected embryological stages from *C. intestinalis*, *L. planeri* and *X. tropicalis* were fixed in RNA*later*™ (Invitrogen, AM7021) for a minimum of 16 h at 4 °C, taking care that the amount of RNA*later*™ was at least 10 times the volume of the sample. Stage numbering was done according to Hotta [35], Tahara [36] and Zahn [37]. RNA extractions were performed with ‘RNAeasy mini kit’ (QIAGEN, 74,104) following the manufacturer’s protocol. The quality and quantity of the total RNA obtained was tested by gel electrophoresis and Nanodrop spectrophotometer.

### cDNA long-read sequencing

For each sample analysed, 50 ng of total RNA was processed using the PCR-cDNA Barcoding Kit (Oxford Nanopore Technologies (ONT), SQK-PCB109). The first strand synthesis was performed following the manufacturer’s instructions (Thermo Scientific Maxima H Minus First Strand cDNA Synthesis Kit with dsDNase, K1681). First, a previous DNAse treatment of the total RNA was performed as follows: incubation of 10 min at 37 °C followed by an inactivation of 5 min at 55 °C in the presence of 10 mM of DTT. After, the sample was cooled on ice and VN Primers and dNTPs where added. After mixing, the sample was incubated 5 min at 65 °C and snap cooled on a pre-chilled freezer block. Then, 5xRT buffer, Strand-Switching Primers and Maxima H Minus Enzyme Mix were added and the sample was mixed by pipetting. The reverse transcription (RT) reaction was performed by incubating the sample 10 min at 25 °C followed by 92 min at 42 °C and 5 min at 85 °C.

A single PCR reaction was performed for each RT reaction. The PCR reaction was prepared according to the PCR-cDNA Barcoding Kit protocol with minor modifications in the cycling conditions: initial denaturation at 95 °C for 30 s; 18 cycles of denaturation at 95 °C for 10 s, annealing at 62 °C for 20 s, extension at 65 °C for 2 min 30 s; final extension of 65 °C for 10 min and hold at 4 °C. Each reaction was treated with Exonuclease I (New England Biolabs, M0293) followed by a purification with AMPure XP beads (Beckman Coulter, A63880) as indicated in the PCR-cDNA Barcoding Kit protocol with minor modification (i.e. we used 30 µL of AMPure XP beads per PCR reaction). The concentration and quality of the obtained samples were assayed by Nanodrop and gel electrophoresis. The sequencing was performed with a maximum of 100 fmol per run.

MinION flow cells (ONT, FLO-MIN106D) underwent flow cell check prior to library construction. The barcoded PCR-cDNA libraries were prepared for sequencing and the MinION flow cell was primed using the flow cell priming kit (ONT, EXP-FLP002) as indicated in the PCR-cDNA Barcoding Kit protocol. A maximum of six PCR-cDNA libraries per run were sequenced in parallel on a single MinION flow cell with Min-KNOWN software v.21.02.2 (ONT). Fast basecalling was performed in real-time with a maximum data acquisition time of 48 h and the following filters applied: minimum Barcode score of 60 and minimum Qscore of 7. All the raw data produced is available at the Sequence Read Archive (SRA) database under accession numbers SRR24756885-SRR24756899, BioProject PRJNA977127.

### Transcriptomic data processing

The cDNA ONT reads were pre-processed with pychopper (ONT), to remove the primer sequences introduced by the protocol, and with SeqKit [38], to remove sequences under 200 bp length. Each cDNA ONT library for each developmental stage sequenced was aligned to the corresponding reference genome by minimap2 [39] (*C. intestinalis* reads aligned against *Ciona robusta* genome (GCA_000224145.2 [40]); *L. planeri* reads aligned against *Petromyzon marinus* genome (GCA_010993605.1 [41]); *X. tropicalis* reads aligned against *X. tropicalis* genome (GCA_000004195.3 [42])), transcripts refined with TranscriptClean [43] and annotated with StringTie2 [44] software. Finally, the stage-specific annotations were merged into a general annotation file for each species with StringTie2 ‘*-merge*’ option and reference transcriptome and proteome dataset were generated using TransDecoder [45]. The obtained proteome datasets and general annotation file were analysed with BUSCO [46] (v5.2.2) and gffcompare [47], respectively, to assess the quality of the transcriptomes. For obtaining the GO annotations, the InterPro Scan option of Blast2GO software (v.6.0.3) and the EggNOG-mapper tool [48] were run using each transcriptome dataset as input.

### Statistical analysis

The GO enrichment analysis was done separately for each species. The analysis was performed in R using the function ‘enricher()’ from the ‘clusterProfiler’ package providing as inputs the list of ‘u’ genes and the GOs and genes found in the whole transcriptome.

For each species dataset, the ratio transcript:gene (t/g ratio) for developmentally expressed genes was calculated for all the genes present in the created transcriptome (All), genes that had more than 1 transcript (Over 1), genes with the gene ontology numbers GO:0009790 (Embryo Development, Emb. Dev.), GO:0016055 (Wnt signalling pathway, Wnt path.), GO:0008101 or GO:0030509 (BMP signalling pathway, BMP path.), GO:0007224 (smoothened signalling pathway, Hh path.) and GO:0003700 (DNA-binding transcription factor activity, TF), and for the gene families SOXs, TCFs, SMADs and GLIs. For the target gene families a BLASTN against the raw reads data was done to ensure isoform detection. We performed *F*-tests (or Bartlett’s test when data not normal by Shapiro Wilk test) to compare the variances of t/g ratios within the different subsets (i.e. All, Over 1, Emb. Dev., Wnt path., BMP path., Hh path., TF, SOXs, TCFs, SMADs and GLIs) doing pairwise comparisons and Bonferroni correction for multiple-test comparisons (Additional File 1: Table S2). A difference in the variance was expected when the t/g ratio was noticeably different within species. The regression coefficient (β_1_) for each subset was estimated using a linear regression model with t/g ratio values and species complexity defined as the number of cell types detected by single-cell RNAseq in larva/organogenesis stage (*C. intestinalis* [49]: 60; *L. planeri* [50]: 70; *X. tropicalis* [51]: 106). Finally, a Mann–Whitney *U* test was applied to evaluate differences in regression coefficients between groups. In all cases, data normality was assessed by Shapiro–Wilk tests (see Additional File 1: Tables S2 and S8).

### RACE-PCR, cloning and sequencing

Total RNA of *C. intestinalis* was used for 3′RACE and 5′RACE experiments using FirstChoice™ RLM-RACE Kit (Invitrogen, AM1700) following the manufacturer’s protocol and with the following CiTCF-specific primers: 5′-CAGGCATGTTACGATACCCATATCCA-3′ (3′RACE), 5′-CATCACAATTTACATCCACATCTGGTGGT-3′ (5′RACE), 5′-CTTCACATATGGCCGACTTGGTTTGTCACCT-3′ (nested 3′RACE) and 5′-TCGCGTTTCTTTGAACCAGGTTCAG-3′ (nested 5′RACE). PCRs were performed with Taq DNA polymerase (Thermo Scientific, EP0402) following the manufacturer’s protocol and results were assessed by gel electrophoresis in 1% agarose gels.

The individual bands obtained after nested RACE-PCR were purified with the ISOLATE II PCR and Gel Kit (Meridian Bioscience, BIO-52059), and cloned with the pGEM®-T Easy Vector System (Promega, A1360), following the manufacturer’s protocol. Transformation was performed into *E. coli* competent cells (Agilent, XL10-Gold Ultracompetent cells, 200,314) by heat-shock. All clones were selected by Ampicillin resistance and their composition was confirmed by enzyme digestion with NotI (New England Biolabs, R0189) and Sanger sequencing (Oxford Zoology service, Eurofins service). The sequences were compared against *C. intestinalis* genomic sequences from Roscoff reference genome (GCA_018327825.1 [52]) and from Plymouth reference genome (GCA_018327805.1 [53]).

The intronic region within exon 12 and exon 13 of *C. intestinalis* TCF was amplified with the following primer pair: 5′-TCGCACGATAATGTTAACAAGC-3′ (forward primer), 5′-GTTCATAGCTACTTGATGGTTGGA-3′ (reverse primer). Specific exon 12.5 PCRs were done with the following primer pair: 5′-ACAACAGCAATTATGGTGCGCAC-3′ (forward primer), 5′- ATACCCCGACGAGGACAAC-3′ (reverse primer). The protocols for PCR and posterior cloning were as described above.

## Supplementary Information

Additional file 1: Tables S1-8. Table S1. GO terms enriched in the novel loci found in our transcriptomic data of Xenopus tropicalis. Table S2. P-values of F-test or Bartlett's tests performed on each group, according to the Shapiro–Wilk test p-values. Table S3. Gffcompare results of X. tropicalis transcriptome, including the GO terms found for each gene ID (qry_gene_id). Table S4. Gffcompare results of L. planeri transcriptome, including the GO terms found for each gene ID (qry_gene_id). Table S5. Gffcompare results of C. intestinalis transcriptome, including the GO terms found for each gene ID (qry_gene_id). Table S6. Linear regression model with individual subsets. The subset 'All' is used as reference for the p-value calculations. Table S7. Linear regression model summaries for each studied subset. Table S8. Regression coefficients obtained from each linear regression performed. Data normality assessed with Shapiro–Wilk test, data homogeneity assessed with Bartlett's test, difference of means assessed with Mann–Whitney U Test. SD: standard deviation.

Additional file 2. Data 1–6. DNA and protein sequences for the TCF/LEF, GLI, and SMAD families found in the transcriptomes reported here.

## Data Availability

All the raw data produced is available at the Sequence Read Archive (SRA) database under accession numbers SRR24756885-SRR24756899, BioProject PRJNA977127. GO terms found for each transcriptome are included in the Additional File 1: Tables S3-5.

## Abbreviations

BLAST: Basic Local Alignment Search Tool
BMP: Bone morphogenetic protein
BUSCO: Benchmarking Universal Single-Copy Orthologs
cDNA: Complementary deoxyribonucleic acid
dNTPs: Deoxynucleotide triphosphates
DTT: Dithiothreitol
GLI: Glioma-Associated Oncogene
GO: Gene Ontology
Hh: Hedgehog
HMG box: High Mobility Group box
MITE: Miniature inverted-repeat transposable elements
RACE PCR: Rapid Amplification of cDNA Ends Polymerase Chain Reaction
RNA: Ribonucleic acid
RT PCR: Reverse transcription polymerase chain reaction
SMAD: Small/Mothers Against DPP Homolog
SOX: SRY-related HMG box
TCF/LEF: T-cell factor/lymphoid enhancer factor
TGFβ: Transforming growth factor β

## References

[1] Adami C. What is complexity? Bioessays. 2002;24:1085–94. 10.1002/bies.10192. 12447974 10.1002/bies.10192

[2] Hahn MW, Wray GA. The g-value paradox. Evol Dev. 2002;4:73–5. 10.1046/j.1525-142X.2002.01069.x. 12004964 10.1046/j.1525-142x.2002.01069.x

[3] Bush SJ, Chen L, Tovar-Corona JM, Urrutia AO. Alternative splicing and the evolution of phenotypic novelty. Philos Trans R Soc Lond B Biol Sci. 2017;372:20150474. 10.1098/rstb.2015.0474. 27994117 10.1098/rstb.2015.0474 PMC5182408

[4] Yang P, Wang D, Kang L. Alternative splicing level related to intron size and organism complexity. BMC Genomics. 2021;22:853. 10.1186/s12864-021-08172-2. 34819032 10.1186/s12864-021-08172-2 PMC8614042

[5] Klingel S, Morath I, Strietz J, Menzel K, Holstein TW, Gradl D. Subfunctionalization and neofunctionalization of vertebrate Lef/Tcf transcription factors. Dev Biol. 2012;368:44–53. 10.1016/j.ydbio.2012.05.012. 22641013 10.1016/j.ydbio.2012.05.012

[6] Marlétaz F, Firbas PN, Maeso I, Tena JJ, Bogdanovic O, Perry M, et al. Amphioxus functional genomics and the origins of vertebrate gene regulation. Nature. 2018;564:64–70. 10.1038/s41586-018-0734-6. 30464347 10.1038/s41586-018-0734-6 PMC6292497

[7] Chen L, Bush SJ, Tovar-Corona JM, Castillo-Morales A, Urrutia AO. Correcting for differential transcript coverage reveals a strong relationship between alternative splicing and organism complexity. Mol Biol Evol. 2014;31:1402–13. 10.1093/molbev/msu083. 24682283 10.1093/molbev/msu083 PMC4032128

[8] Hoppler S, Moon RT, editors. Wnt Signaling in Development and Disease. Hoboken, NJ, USA: John Wiley & Sons, Inc; 2014. 10.1002/9781118444122.

[9] Steinhart Z, Angers S. Wnt signaling in development and tissue homeostasis. Development. 2018. 10.1242/dev.146589. 29884654 10.1242/dev.146589

[10] Mayer C-D, de La Giclais SM, Alsehly F, Hoppler S. Diverse LEF/TCF Expression in Human Colorectal Cancer Correlates with Altered Wnt-Regulated Transcriptome in a Meta-Analysis of Patient Biopsies. Genes (Basel). 2020;11:538. 10.3390/genes11050538. 32403323 10.3390/genes11050538 PMC7288467

[11] Pradas-Juni M, Nicod N, Fernández-Rebollo E, Gomis R. Differential transcriptional and posttranslational transcription factor 7-like 2 regulation among nondiabetic individuals and type 2 diabetic patients. Mol Endocrinol. 2014;28:1558–70. 10.1210/me.2014-1065. 25058603 10.1210/me.2014-1065 PMC5414799

[12] Bem J, Brożko N, Chakraborty C, Lipiec MA, Koziński K, Nagalski A, et al. Wnt/β‐catenin signaling in brain development and mental disorders: keeping TCF7L2 in mind. FEBS Lett. 2019;593:1654–74. 10.1002/1873-3468.13502. 31218672 10.1002/1873-3468.13502 PMC6772062

[13] Akiyama T, Raftery LA, Wharton KA. Bone morphogenetic protein signaling: the pathway and its regulation. Genetics. 2023;226:iyad200. 10.1093/genetics/iyad200. 10.1093/genetics/iyad200 PMC10847725 38124338

[14] Jing J, Wu Z, Wang J, Luo G, Lin H, Fan Y, et al. Hedgehog signaling in tissue homeostasis, cancers and targeted therapies. Signal Transduct Target Ther. 2023;8:315. 10.1038/s41392-023-01559-5. 37596267 10.1038/s41392-023-01559-5 PMC10439210

[15] Xu J, Iyyanar PPR, Lan Y, Jiang R. Sonic hedgehog signaling in craniofacial development. Differentiation. 2023;133:60–76. 10.1016/j.diff.2023.07.002. 37481904 10.1016/j.diff.2023.07.002 PMC10529669

[16] Croce JC, Holstein TW. The Wnt’s Tale. In: Wnt Signaling in Development and Disease. Hoboken, NJ, USA: John Wiley & Sons, Inc; 2014. p. 161–76. 10.1002/9781118444122.ch12.

[17] Hoppler S, Kavanagh CL. Wnt signalling: variety at the core. J Cell Sci. 2007;120:385–93. 10.1242/jcs.03363. 17251379 10.1242/jcs.03363

[18] Mao CD, Byers SW. Cell-context dependent TCF/LEF expression and function: alternative tales of repression, de-repression and activation potentials. Crit Rev Eukaryot Gene Expr. 2011;21:207–36. 10.1615/CritRevEukarGeneExpr.v21.i3.10. 22111711 10.1615/critreveukargeneexpr.v21.i3.10 PMC3434703

[19] Cadigan KM, Waterman ML. TCF/LEFs and Wnt signaling in the nucleus. Cold Spring Harb Perspect Biol. 2012;4:a007906–a007906. 10.1101/cshperspect.a007906. 23024173 10.1101/cshperspect.a007906 PMC3536346

[20] Hoppler S, Waterman ML. Evolutionary Diversification of Vertebrate TCF/LEF Structure, Function, and Regulation. In: Wnt Signaling in Development and Disease. Hoboken, NJ, USA: John Wiley & Sons, Inc; 2014. p. 225–37. 10.1002/9781118444122.ch17.

[21] Torres‐Aguila NP, Salonna M, Hoppler S, Ferrier DEK. Evolutionary diversification of the canonical Wnt signaling effector TCF/LEF in chordates. Dev Growth Differ. 2022;64:120–137. 10.1111/dgd.12771. 10.1111/dgd.12771 PMC9303524 35048372

[22] Garstang MG, Osborne PW, Ferrier DEK. TCF/Lef regulates the Gsx parahox gene in central nervous system development in chordates. BMC Evol Biol. 2016;16:57. 10.1186/s12862-016-0614-3. 26940763 10.1186/s12862-016-0614-3 PMC4776371

[23] Simmen MW, Bird A. Sequence analysis of transposable elements in the sea squirt, *Ciona intestinalis*. Mol Biol Evol. 2000;17:1685–94. 10.1093/oxfordjournals.molbev.a026267. 11070056 10.1093/oxfordjournals.molbev.a026267

[24] Dehal P, Satou Y, Campbell RK, Chapman J, Degnan B, De Tomaso A, et al. The draft genome of *Ciona intestinalis* : insights into chordate and vertebrate origins. Science. 2002;298:2157–67. 10.1126/science.1080049. 12481130 10.1126/science.1080049

[25] Attisano L, Tuen Lee-Hoeflich S. The smads. Genome Biol. 2001;2:reviews3010.1. 10.1186/gb-2001-2-8-reviews3010. 11532220 10.1186/gb-2001-2-8-reviews3010 PMC138956

[26] Massagué J, Seoane J, Wotton D. Smad transcription factors. Genes Dev. 2005;19:2783–810. 10.1101/gad.1350705. 16322555 10.1101/gad.1350705

[27] Hooper JE, Scott MP. Communicating with hedgehogs. Nat Rev Mol Cell Biol. 2005;6:306–17. 10.1038/nrm1622. 15803137 10.1038/nrm1622

[28] Sigafoos AN, Paradise BD, Fernandez-Zapico ME. Hedgehog/GLI signaling pathway: transduction, regulation, and implications for disease. Cancers (Basel). 2021;13:3410. 10.3390/cancers13143410. 34298625 10.3390/cancers13143410 PMC8304605

[29] Weise A, Bruser K, Elfert S, Wallmen B, Wittel Y, Wohrle S, Hecht A. Alternative splicing of Tcf7l2 transcripts generates protein variants with differential promoter-binding and transcriptional activation properties at Wnt/beta-catenin targets. Nucleic Acid Res 2010;38:1964–1981. 10.1093/nar/gkp1197. 10.1093/nar/gkp1197 PMC2847248 20044351

[30] Kopelman NM, Lancet D, Yanai I. Alternative splicing and gene duplication are inversely correlated evolutionary mechanisms. Nat Genet. 2005;37:588–9. 10.1038/ng1575. 15895079 10.1038/ng1575

[31] MacLean DW, Meedel TH, Hastings KEM. Tissue-specific alternative splicing of ascidian troponin I isoforms. J Biol Chem. 1997;272:32115–20. 10.1074/jbc.272.51.32115. 9405409 10.1074/jbc.272.51.32115

[32] Holland LZ. Genomics, evolution and development of amphioxus and tunicates: the Goldilocks principle. J Exp Zool B Mol Dev Evol. 2015;324:342–52. 10.1002/jez.b.22569. 24665055 10.1002/jez.b.22569

[33] Huang X, Ren Q, Wang Y, Shimeld SM, Li G. Amphioxus Gli knockout disrupts the development of left–right asymmetry but has limited impact on neural patterning. Mar Life Sci Technol. 2023;5:492–9. 10.1007/s42995-023-00195-w. 38045549 10.1007/s42995-023-00195-w PMC10689630

[34] Smith JJ, Kuraku S, Holt C, Sauka-Spengler T, Jiang N, Campbell MS, et al. Sequencing of the sea lamprey (*Petromyzon marinus*) genome provides insights into vertebrate evolution. Nat Genet. 2013;45:415–21. 10.1038/ng.2568. 23435085 10.1038/ng.2568 PMC3709584

[35] Hotta K, Mitsuhara K, Takahashi H, Inaba K, Oka K, Gojobori T, et al. A web-based interactive developmental table for the ascidian *Ciona intestinalis*, including 3D real-image embryo reconstructions: I. From fertilized egg to hatching larva. Dev Dyn. 2007;236:1790–805. 10.1002/dvdy.21188. 17557317 10.1002/dvdy.21188

[36] Tahara Y. Normal stages of development in the lamprey, *Lampetra reissneri* (Dybowski). Zoolog Sci. 1988;5:109–18.

[37] Zahn N, Levin M, Adams DS. The Zahn drawings: new illustrations of *Xenopus* embryo and tadpole stages for studies of craniofacial development. Development. 2017;144:2708–13. 10.1242/dev.151308. 28765211 10.1242/dev.151308 PMC5560046

[38] Shen W, Le S, Li Y, Hu F. Seqkit: a cross-platform and ultrafast toolkit for FASTA/Q file manipulation. PLoS One. 2016;11:e0163962. 10.1371/journal.pone.0163962. 27706213 10.1371/journal.pone.0163962 PMC5051824

[39] Li H. New strategies to improve minimap2 alignment accuracy. Bioinformatics. 2021;37:4572–4. 10.1093/bioinformatics/btab705. 34623391 10.1093/bioinformatics/btab705 PMC8652018

[40] Department of Zoology, Graduate School of Science, Kyoto University. Ciona intestinalis KH genome assembly (GCA_000224145.2). NCBI Datasets. 2013. https://identifiers.org/ncbi/assembly:GCA_000224145.2.

[41] Vertebrate Genomes Project. Petromyzon marinus (kPetMar1) genome assembly. NCBI Datasets. 2020. https://identifiers.org/ncbi/assembly:GCA_010993605.1.

[42] DOE Joint Genome Institute. Xenopus tropicalis (Xenopus_tropicalis_v9.1) genome assembly (GCA_000004195.3). NCBI Datasets. 2016. https://identifiers.org/ncbi/assembly:GCA_000004195.3.

[43] Wyman D, Mortazavi A. Transcriptclean: variant-aware correction of indels, mismatches and splice junctions in long-read transcripts. Bioinformatics. 2019;35:340–2. 10.1093/bioinformatics/bty483. 29912287 10.1093/bioinformatics/bty483 PMC6329999

[44] Kovaka S, Zimin V., Pertea GM, Razaghi R, Salzberg SL, Pertea M. Transcriptome assembly from long-read RNA-seq alignments with StringTie2. Genome Biol. 2019;20:278. 10.1186/s13059-019-1910-1. 31842956 10.1186/s13059-019-1910-1 PMC6912988

[45] Haas BJ. TransDEcoder. https://github.com/TransDecoder/TransDecoder.

[46] Manni M, Berkeley MR, Seppey M, Simão FA, Zdobnov EM. BUSCO update: novel and streamlined workflows along with broader and deeper phylogenetic coverage for scoring of eukaryotic, prokaryotic, and viral genomes. Mol Biol Evol. 2021;38:4647–54. 10.1093/molbev/msab199. 34320186 10.1093/molbev/msab199 PMC8476166

[47] Pertea G, Pertea M. GFF utilities: GffRead and GffCompare. F1000Res. 2020;9:304. 10.12688/f1000research.23297.1. 10.12688/f1000research.23297.1 PMC7222033 32489650

[48] Cantalapiedra CP, Hernández-Plaza A, Letunic I, Bork P, Huerta-Cepas J. eggNOG-mapper v2: functional annotation, orthology assignments, and domain prediction at the metagenomic scale. Mol Biol Evol. 2021;38:5825–9. 10.1093/molbev/msab293. 34597405 10.1093/molbev/msab293 PMC8662613

[49] Cao C, Lemaire LA, Wang W, Yoon PH, Choi YA, Parsons LR, et al. Comprehensive single-cell transcriptome lineages of a proto-vertebrate. Nature. 2019;571:349–54. 10.1038/s41586-019-1385-y. 31292549 10.1038/s41586-019-1385-y PMC6978789

[50] Pang Y, Qin Y, Du Z, Liu Q, Zhang J, Han K, et al. Single-cell transcriptome atlas of lamprey exploring Natterin- induced white adipose tissue browning. Nat Commun. 2025;16:752. 10.1038/s41467-025-56153-w. 39820434 10.1038/s41467-025-56153-w PMC11739602

[51] Liao Y, Ma L, Guo Q, E W, Fang X, Yang L, et al. Cell landscape of larval and adult *Xenopus laevis* at single-cell resolution. Nat Commun. 2022;13:4306. 10.1038/s41467-022-31949-2. 35879314 10.1038/s41467-022-31949-2 PMC9314398

[52] Marine Genomics Unit, Okinawa Institute of Science and Technology. Ciona intestinalis (Cint(typeB-Roscoff)_1.0) genome assembly. NCBI Datasets. 2021. https://identifiers.org/ncbi/assembly:GCA_018327825.1.

[53] Marine Genomics Unit, Okinawa Institute of Science and Technology. Ciona intestinalis (Cint(typeB-Plymouth)_1.0) genome assembly. NCBI Datasets. 2021. https://identifiers.org/ncbi/assembly:GCA_018327805.1.

